# Improving the Gastrointestinal Stability of Linaclotide

**DOI:** 10.1021/acs.jmedchem.1c00380

**Published:** 2021-05-12

**Authors:** Nayara Braga Emidio, Hue N. T. Tran, Asa Andersson, Philip E. Dawson, Fernando Albericio, Irina Vetter, Markus Muttenthaler

**Affiliations:** † Institute for Molecular Bioscience, The University of Queensland, Brisbane, Queensland 4072, Australia; ‡ Department of Chemistry, The Scripps Research Institute, La Jolla, California 92037, United States; § CIBER-BBN, Networking Centre on Bioengineering, Biomaterials and Nanomedicine, and Department of Organic Chemistry, University of Barcelona, 08028 Barcelona, Spain; ∥ School of Pharmacy, Pharmacy Australia Centre of Excellence, The University of Queensland, Woolloongabba, QLD 4102, Australia; ⊥ Institute of Biological Chemistry, Faculty of Chemistry, University of Vienna, 1090 Vienna, Austria

## Abstract

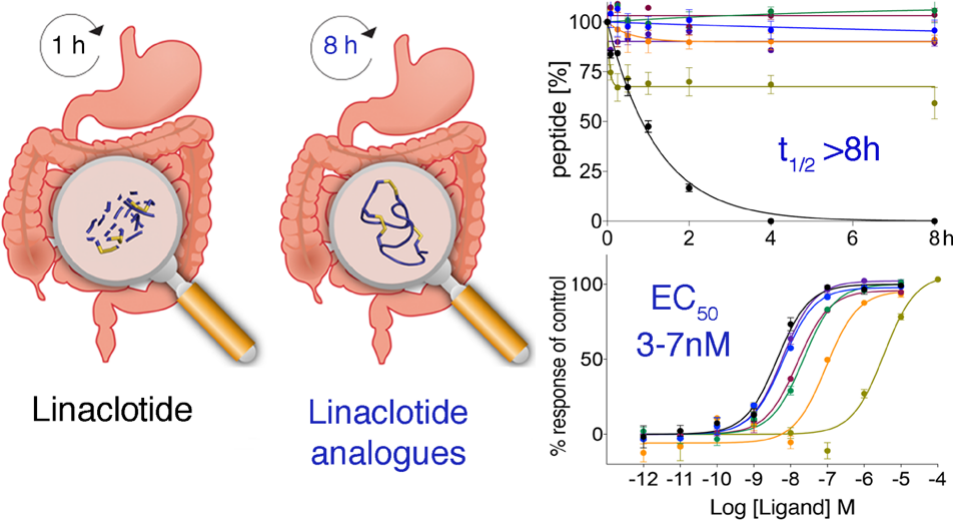

High susceptibility to proteolytic degradation in the gastrointestinal tract limits the therapeutic application of peptide drugs in gastrointestinal disorders. Linaclotide is an orally administered peptide drug for the treatment of irritable bowel syndrome with constipation (IBS-C) and abdominal pain. Linaclotide is however degraded in the intestinal environment within 1 h, and improvements in gastrointestinal stability might enhance its therapeutic application. We therefore designed and synthesized a series of linaclotide analogues employing a variety of strategic modifications and evaluated their gastrointestinal stability and pharmacological activity at its target receptor guanylate cyclase-C. All analogues had substantial improvements in gastrointestinal half-lives (>8 h vs linaclotide 48 min), and most remained active at low nanomolar concentrations. This work highlights strategic approaches for the development of gut-stable peptides toward the next generation of orally administered peptide drugs for the treatment of gastrointestinal disorders.

## Introduction

Linaclotide is an orally administered peptide drug approved by the Food and Drug Administration (FDA) in 2012 for the treatment of irritable bowel syndrome with constipation (IBS-C) and abdominal pain.^1,2^ Linaclotide elicits a local pharmacological response in the gastrointestinal tract by activating the guanylate cyclase-C (GC-C), a receptor predominantly expressed on the luminal surface of epithelial cells throughout the intestine. Stimulation of GC-C results in accumulation of intracellular levels of the downstream effector cyclic-guanosine-3′,5′-monophosphate (cGMP).^3^ Increased cGMP levels stimulate the secretion of water and electrolytes into the intestinal lumen, which accelerates the gastrointestinal transit and resolves constipation; it also inhibits colonic nociceptors, thereby reducing abdominal pain.^1−3^

**Figure 1:**
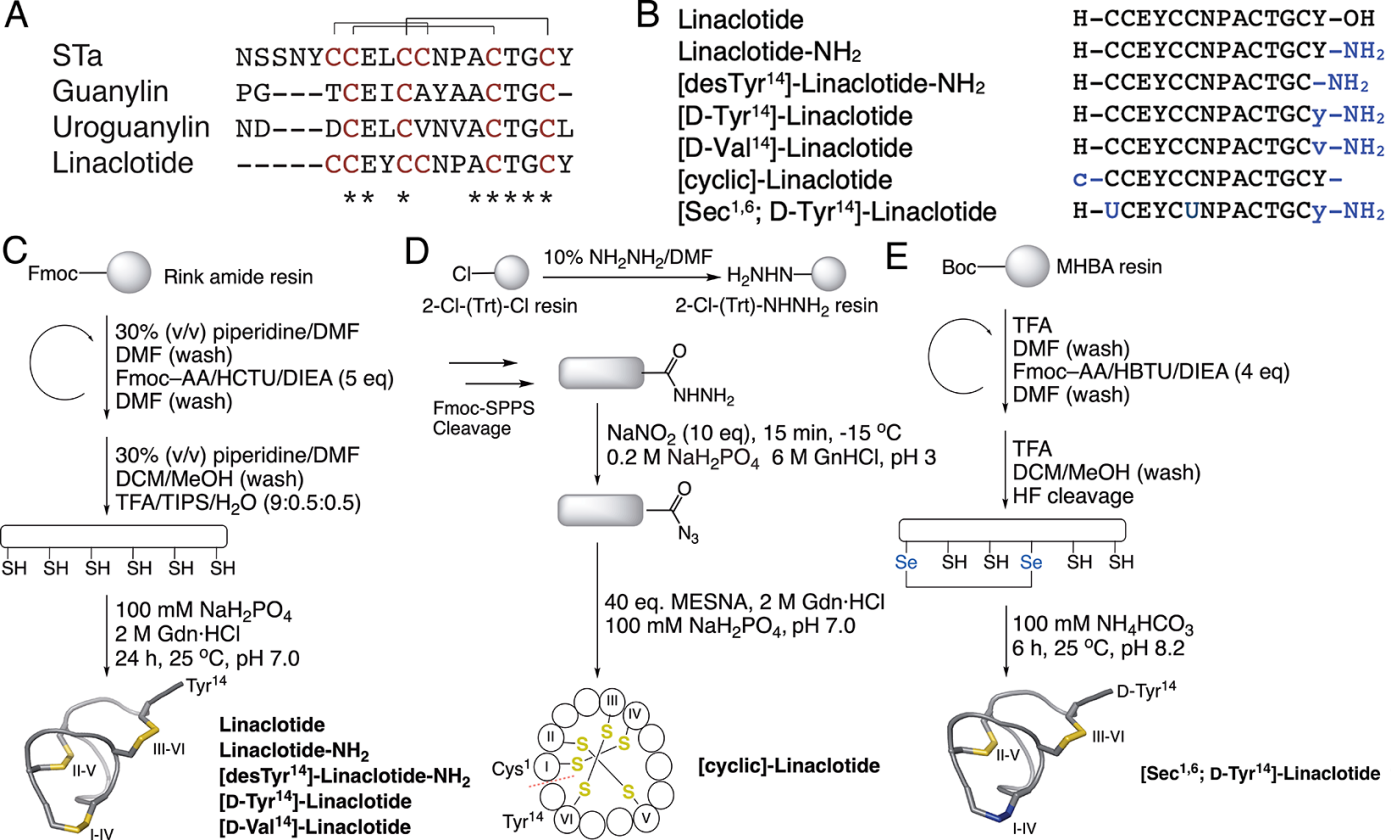
Design and synthesis of linaclotide and its analogues. (A) Linaclotide is a hybrid design of bacterial heat-stable enterotoxin (STa) and endogenous peptide hormones guanylin and uroguanylin. (B) Sequence of linaclotide and its analogues. Modifications are highlighted in blue; c/- backbone cyclization; y: d-Tyr; v: d-Val; U: selenocysteine (C) Peptides were obtained by Fmoc-SPPS followed by oxidative folding. (D) Fresh 2-chlorotrityl hydrazine resin was prepared to synthesize the peptides *via* Fmoc-SPPS followed by one-pot cyclization *via* intramolecular hydrazide-based native chemical ligation (NCL) and oxidative folding. The dashed line indicates the ligation site. (E) [Sec^1,6^; d-Tyr^14^]-Linaclotide was synthesized *via* Boc-SPPS using Boc-Sec(Meb)-OH to introduce the two selenocysteine residues followed by HF cleavage and oxidative folding.

Linaclotide is a hybrid design of a bacterial heat-stable enterotoxin (STa) that causes diarrhea and the endogenous peptide hormones guanylin and uroguanylin.^4−7^ STa potently activates GC-C and is 10 and 100 times more potent than uroguanylin and guanylin, respectively.^8^ Linaclotide is 14 amino acid residues long and is a designed hybrid of these three peptides (Figure 1A). Similar to STa, linaclotide holds three disulfide bonds in a Cys^I^-Cys^IV^, Cys^II^-Cys^V^, and Cys^III^-Cys^VI^ connectivity, while uroguanylin and guanylin only have two disulfide bonds. The three-disulfide bond arrangement stabilizes three β-turns and locks the molecule into its active conformation while conferring enhanced stability compared to the endogenous peptides.^3,9^

Typically, orally administered peptides are rapidly degraded by proteases in the gut, limiting their therapeutic use for gastrointestinal disorders. Linaclotide is however stable in the gastric environment for at least 3 h^3^ and stable enough in the intestinal environment to elicit a therapeutic response. The therapeutic response is actually driven by the GC-C-active metabolite MM-419447, which is rapidly produced upon the cleavage of linaclotide’s *C*-terminal Tyr^14^.^10^*In vitro*, both linaclotide and MM-419447 are degraded within 1 h in simulated intestinal conditions, starting with the reduction of their disulfide bonds.^10^ Linaclotide absorption into the systemic circulation is insignificant, and only small amounts (3–5%) of linaclotide or MM-419447 are excreted in the feces, supporting the fact that their degradation also happens *in vivo*.^10^

Effective pharmacotherapy depends on the local concentration of linaclotide or MM-419447.^10^ Enhancing the stability of linaclotide in the gastrointestinal tract has therefore the potential to decrease the administered dose and improve the therapeutic applications of this innovative peptide drug. Hence, we explored a range of rational modifications (Figure 1B) to enhance the gastrointestinal stability of linaclotide while retaining its pharmacological activity. Such strategies should be highly valuable and applicable also for other peptides with therapeutic potential in gastrointestinal disorders and could lead to more orally administered peptide therapeutics and better treatment options.

## Results

### Design of Gut-Stable Linaclotide Analogues

Oral administration of peptides is usually hampered by rapid degradation in the gastrointestinal tract. The gut is a hostile milieu for peptides, where they are exposed to acidic pH, a variety of proteases that cleave susceptible amino acids, and a large number of bacteria that secrete metabolic enzymes.^11,12^ Here, we designed a series of linaclotide analogues (Figure 1) to study a range of chemical modifications in terms of gastrointestinal stability and their ability to activate the GC-C receptor. Considering linaclotide’s stability data,^3,9,10^ we particularly focused on stabilizing the *C*-terminal and the disulfide bonds.

Linaclotide has a *C*-terminal acid and Tyr at position 14, which is readily cleaved in the intestine, producing the GC-C-active metabolite MM-419447, again with a *C*-terminal acid. *C*-terminal acids, however, have a negative charge that is readily recognized by carboxypeptidases. It is thus no surprise that, in nature, more than half of the biologically active peptides have a post-translationally modified *C*-terminal amide, which provides improvement in stability due to the lack of negative charge.^13−16^ Therefore, our first step was to produce the *C*-terminal amide analogue (Linaclotide-NH_2_) as well as an amidated version of MM-419447 ([desTyr^14^]-Linaclotide-NH_2_).

Replacement of levorotatory l-amino acids by dextrorotatory d-amino acids enhances metabolic resistance against proteases since d-amino acids are rarely recognized and cleaved by proteases.^17^ Strategic placement of a d-amino acid at its *N*- or *C*-terminus can have a substantial impact on the metabolic stability of a peptide since it often prevents the first step of enzymatic degradation. We therefore designed and synthesized [d-Tyr^14^]-Linaclotide. We also wanted to know if position 14 could be replaced by non-tyrosine residues; hence, we included [d-Val^14^]-Linaclotide in our series. This information could become useful for the design of gut-stable GC-C probes with *C*-terminal reporter tags.

*N*-to-*C*-terminal backbone cyclization is another strategy that has received much attention in improving a peptide’s bioactivity and metabolic stability by constraining its conformational flexibility.^18−23^ We therefore included a backbone cyclized analogue ([cyclic]-Linaclotide) in our structure–activity relationship (SAR) study.

Finally, disulfide bolds can be reduced in the gastrointestinal environment, and much work has been carried out to develop more stable disulfide bond mimetics.^24−29^ The diselenide bond is one of the most conservative substitutions while providing enhanced protection against reduction due to its lower redox potential.^26−34^ Substitution of a single disulfide bond by a diselenide bond is sufficient to avoid scrambling or reduction in reducing conditions, thereby deactivating the peptide.^28,33^ Given that the cleavage of Tyr^14^ and reduction of the disulfide bonds are the first steps in the degradation of linaclotide and MM-419447,^10^ we designed [Sec^1,6^; d-Tyr^14^]-Linaclotide.

### Peptide Synthesis

Peptides were obtained by Fmoc-SPPS (9-fluorenymethyloxycarbonyl-solid phase peptide synthesis) followed by oxidative folding (Figure 1C) or by Fmoc-SPPS in combination with one-pot cyclization *via* intramolecular hydrazide-based native chemical ligation (NCL) and oxidative folding (Figure 1D). Oxidative folding, carried out in 100 mM NaH_2_PO_4_, 2 M Gdn·HCl, pH 7.0, yielded one predominant isomer in all analogues, as reported for linaclotide.^35^ All analogues were generated in good purity and quantity (>95% purity, >10% overall yield), except for [cyclic]-Linaclotide, which had a 4% overall yield (>95% purity) (Figure S1). [Sec^1,6^; d-Tyr^14^]-Linaclotide was synthesized *via tert*-butyloxycarbonyl (Boc) SPPS using Boc-Sec(Meb)-OH, as described previously (Figure 1E).^28^

### *In Vitro* Gastrointestinal Stability

**Figure 2:**
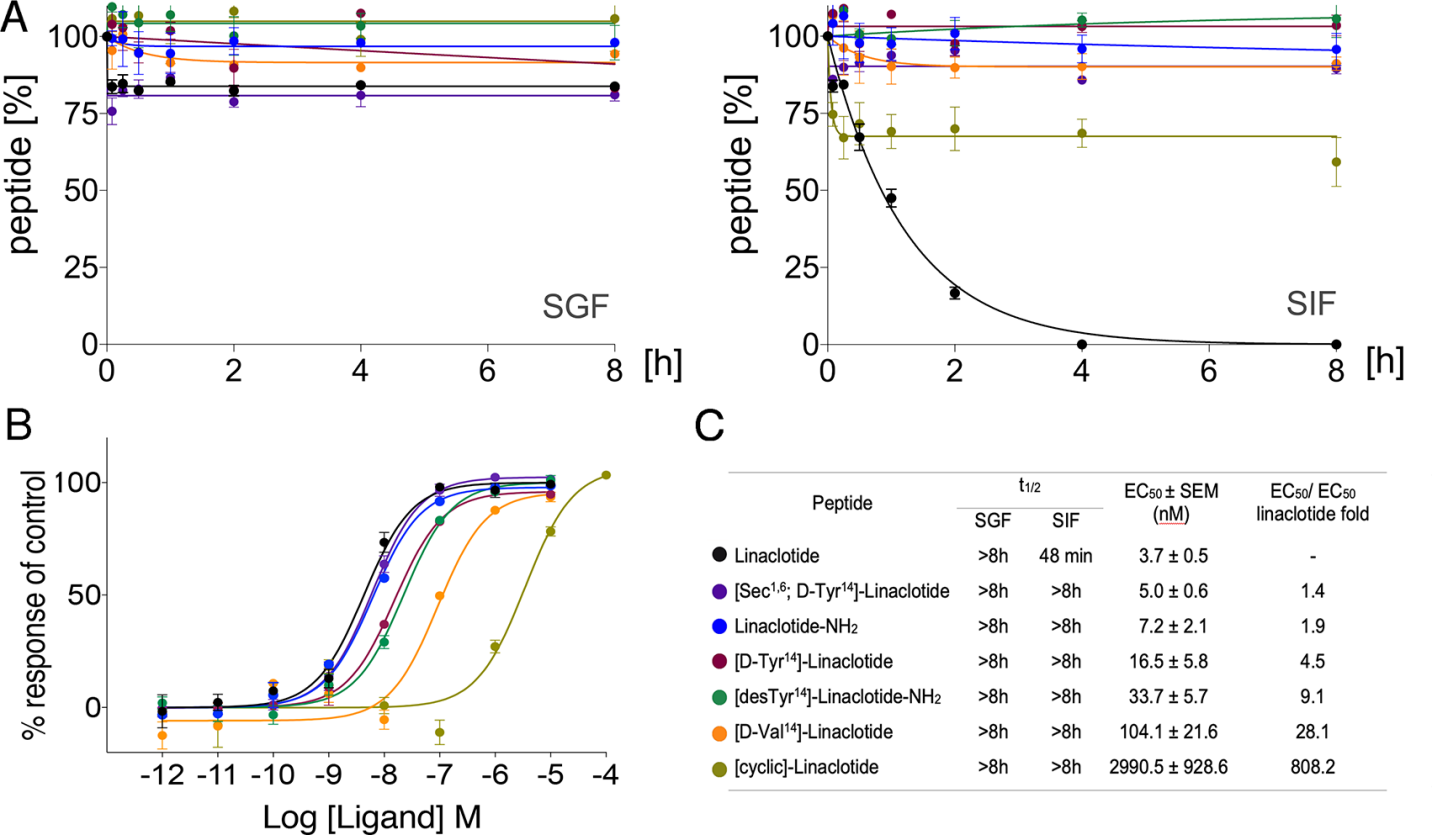
Gastrointestinal stability and pharmacological characterization of linaclotide and its modified analogues. (A) Gastrointestinal stability assays were carried out using simulated gastric fluid (SGF) and simulated intestinal fluid (SIF). Results are expressed (mean ± SEM) as the percentage of the area under the peak (analytical C_18_-RP-HPLC, 214 nm) at each time point to that of *t* = 0 h (*n* ≥ 3). Curves were fit to the data points using a one-phase decay. (B) Representative concentration response of cGMP accumulation in human epithelial intestinal T84 cells. Data are presented as mean ± SEM (*n* ≥ 3). Curves were fit to the data using a four-parameter Hill equation with a variable Hill coefficient (GraphPad Prism 7.0). (C) Summary of the half-lives in SGF and SIF and EC_50_ and EC_50_/EC_50_ linaclotide fold.

We assessed the gastrointestinal stability of our linaclotide analogues in well-established simulated gastric (SGF) and intestinal fluid (SIF) stability assays that mimic the human physiological conditions in the stomach and intestine.^36,37^ Our modifications all resulted in substantially improved intestinal half-lives (*t*_1/2_ = >8 h) compared to linaclotide (*t*_1/2_ = 48 min) (Figure 2A,C). [cyclic]-Linaclotide was the least stable of the newly designed analogues, and we did not observe a stable metabolite in the SIF, indicating a different degradation pathway as for linaclotide.

### cGMP Accumulation in Human T84 Cells

We evaluated our linaclotide analogues for their ability to activate the GC-C receptor in human epithelial intestinal T84 cells that natively express this receptor. GC-C activation results in production of second messenger cGMP, which was measured using a commercially available kit (cGMP HTRF assay kit, Cisbio International). As a control, we tested the concentration of linaclotide (3.7 ± 0.5 nM) required to induce 50% of the maximal activity (EC_50_). All analogues, except [cyclic]-Linaclotide, retained the ability to increase cGMP levels at nanomolar concentrations (Figure 2B,C). Linaclotide-NH_2_ (7.2 ± 2.1 nM) displayed similar potency as linaclotide (3.7 ± 0.5 nM). Interestingly, so did [Sec^1,6^; d-Tyr^14^]-Linaclotide (5.0 ± 0.6 nM), even though d-Tyr-linaclotide (16.5 ± 5.8 nM) was 4.5-fold less potent than linaclotide. d-Val^14^-Linaclotide (104.1 ± 21.6 nM) displayed a 28-fold reduced potency, indicating that the *C*-terminus is not entirely modifiable without impacting GC-C activation, a trend also observed for [desTyr^14^]-Linaclotide-NH_2_ (33.7 ± 5.7 nM) and even further pronounced through *N*-to-*C*-terminal cyclization in [cyclic]-Linaclotide, where we observed an 808-fold lower activity (2990.5 ± 928.6 nM) (Figure 2B,C).

## Discussion

Developing gut-stable peptides for therapeutic applications in the gut is a new and highly innovative direction to address a main disadvantage of peptide drugs, namely, their route of administration (>90% of peptide drugs have to be injected). Linaclotide is a front runner in a new class of oral peptide drugs that target receptors accessible in the gastrointestinal lumen to elicit a therapeutic response. Even though linaclotide is more stable against chymotrypsin than the endogenous GC-C ligand guanylin, it is still degraded rapidly in the intestine.^10,38^ Linaclotide has an extra disulfide bond compared to guanylin, which constrains the peptide in its active conformation and enhances its stability and potency.^3,9,10^ In contrast, uroguanylin and guanilyn form two interchangeable topoisomers with different affinities toward the GC-C receptor.^39−42^

Cleavage of Tyr^14^ and disulfide bond reduction are the first steps in the gastrointestinal breakdown of linaclotide, leading to inactivation of both linaclotide and its metabolite.^10^ We thus hypothesized that protecting the *C*-terminus from exopeptidases *via C*-terminal amidation, introduction of a d-amino acid, or *N*-to-*C*-terminal backbone cyclization would improve the gastrointestinal stability, and we also explored a diselenide mimetic to prevent disulfide bond scrambling and reduction (Figure 1). These subtle and strategic modifications resulted in highly stable analogues with intestinal half-lives of more than 8 h compared to 48 min of linaclotide.

The modifications were overall well tolerated and resulted in analogues nearly equipotent to linaclotide (Figure 2). Simple *C*-terminal amidation of linaclotide or its metabolite, a strategy often observed in nature,^13−16^ enhanced gastrointestinal stability substantially while retaining low nanomolar potency at the GC-C receptor. This modification, along with introduction of d-Tyr^14^, is a simple approach to produce potent and gut-stable linaclotide analogues. [Sec^1,6^; d-Tyr^14^]-Linaclotide is an interesting analogue since the diselenide in positions 1 and 6 rescued some of the potency loss of [d-Tyr^14^]-Linaclotide, being nearly equally potent as linaclotide with the advantage that the diselenide bond is harder to reduce.^28,33^

Linaclotide and MM-419447 are equipotent;^10^ however, amidation of MM-419447 resulted in an analogue 9-fold less potent than linaclotide/MM-419447. Introduction of d-Val^14^ (28-fold less potent than linaclotide and 6-fold less than [d-Tyr^14^]-Linaclotide) had an impact on activity. Together, these findings suggest that care needs to be taken when modifying position 14. [d-Val^14^]-Linaclotide still had an EC_50_ of 104 nM and was gut-stable, suggesting that the introduction of reporter tags such as fluorophores or biotin at the *C*-terminal could lead to gut-stable molecular probes useful for studying the GC-C receptor in the gut or the pharmacokinetics/dynamics of linaclotide. *N*-to-*C*-terminal backbone cyclization, even though it provided another gut-stable analogue, was not well tolerated in terms of bioactivity (∼800-fold less potent than linaclotide). Considering the distance between the *N*- and *C*-termini in linaclotide,^3,9^ backbone cyclization could over-constrain the peptide resulting in conformational changes or misfolding that affect activity. Comparison of the 1D ^1^H NMR spectra in aqueous solution confirmed this, showing clear differences in the dispersion of the chemical shifts of [cyclic]-Linaclotide (some chemical shifts are poorly dispersed leading to broad peaks) compared to linaclotide and other analogues (good chemical shift dispersion and sharp peaks) (Figures S2 and S3). For the design of novel linaclotide analogues with backbone cyclization, one might consider the use of cyclization linkers and a directed folding approach.^18,23^ However, given the lack of activity and great gut stability, [cyclic]-Linaclotide could become useful as a biologically inert and gut-stable scaffold for grafting peptide sequences into its scaffold, similarly as it has been done for cyclotides and sunflower trypsin inhibitor 1 (STF-1).^23,43^

Linaclotide analogues with improved gastrointestinal stability could lead to better therapeutics than the front-runner linaclotide. No degradation means fewer metabolites, which could be responsible for observed side effects such as dose-dependent diarrhea, abdominal discomfort, and flatulence.^44,45^ Higher stability is also linked to lower doses required, which could provide overall better treatment options at lower costs.

## Conclusions

We demonstrated that subtle but strategic modifications to linaclotide can yield bioactive gut-stable analogues, exemplifying the concept of developing gut-stable peptide therapeutics that can be orally administered. In particular, amidated linaclotide is of interest where a conservative chemical modification (CONH_2_ instead of COOH) resulted in a substantially more stable analogue with potent activity. Gut-stable GC-C agonists are expected to result in more prolonged effects and fewer side effects due to cleaner pharmacodynamics since no metabolites are produced. Gut-stable peptides targeting accessible receptors in the lumen of the gastrointestinal tract are a promising new class of oral peptide therapeutics that elegantly address the problem of low patient compliance and acceptance of injectable peptide drugs. This concept of orally administered (but not orally bioavailable) peptide drugs could become a game changer for gastrointestinal disorders, where gut peptides, immune host defense peptides, and antimicrobial/anti-biofilm peptides often form the first host response to restore gastrointestinal homeostasis after an infection or injury.

## Experimental Section

### Materials

Fmoc-protected amino acid building blocks were purchased from Iris Biotech GmbH (Marktredwitz, Germany). 2-Chlorotrityl chloride resin (loading 1.0–2.0 mmol/g) and *O*-(1*H*-6-chlorobenzotriazol-1-yl)-*N*,*N*,*N*′,*N*′-tetramethyluronium hexafluorophosphate (HCTU) were purchased from Chem-Impex (Wood Dale, USA). Rink amide resin (loading 0.74 mmol/g) was from RAPP Polymere (Tuebingen, Germany). *N*,*N*-Diisopropylethylamine (DIPEA) peptide synthesis grade was from Auspep (Melbourne, Australia). Hydrazine hydrate, tri-isopropylsilane (TIPS), acetonitrile (ACN), sodium 2-mercaptoethanesulfonate (MESNA), tris(2-carboxyethyl)phosphine (TCEP), pepsin (3500–4500 U/mg), and reduced l-glutathione were from Sigma-Aldrich (Sydney, Australia). *N*,*N*-Dimethylformamide (DMF), trifluoroacetic acid (TFA), porcine pancreatin, and diethyl ether were obtained from Chem-Supply (Gillman, Australia). Trypsin-EDTA (0.25%), Dulbecco’s modified Eagle’s medium (DMEM), and l-glutamine were from Invitrogen (Mulgrave, Australia). Fetal bovine serum (FBS) was from Scientifix (South Yarra, Australia). The cGMP assay kit was from Cisbio (Bedford, USA). The HT-84 cell line was obtained from CellBank Australia (Wentworthville, Australia). Dulbecco’s modified Eagle’s medium (DMEM) and Ham’s F-12 medium (1:1) were obtained from Thermo Fisher Scientific (Australia). All other reagents and solvents were obtained from Sigma-Aldrich (Sydney, Australia) in the highest available purity and used without further purification.

### Solid Phase Peptide Synthesis

Peptides were synthesized on a Symphony automated peptide synthesizer (Protein Technologies Inc., Tucson, AZ) *via* Fmoc-SPPS on a 0.1 mmol scale using Rink amide resin (RAM; RAPP Polymere, 0.74 mmol/g) or freshly prepared 2-chlorotrityl hydrazine resin. Fmoc deprotection was achieved using 30% (v/v) piperidine/DMF (2 × 5 min). Couplings were carried out in DMF using 5 equiv relative to the resin loading of Fmoc–amino acid acid/HCTU/DIEA (1:1:1) twice (5 and 10 min). Amino acid side chains were protected as follows: Asn(Trt), Glu(OtBu), Cys(Trt), Lys, and Thr/Tyr(tBu). The simultaneous peptide cleavage from the resin and removal of side-chain protecting groups were carried out using 90% TFA/5% TIPS/5% H_2_O for 2 h at 25 °C. Cleavage solution was evaporated using N_2_. The peptides were precipitated and washed with diethyl ether three times and then lyophilized in 50% ACN/0.1% TFA/H_2_O. [Sec^1,6^; d-Tyr^14^]-Linaclotide was synthesized *via* Boc-SPPS using Boc-Sec(Meb)-OH, as described previously.^28^

### Oxidative Folding

Peptides were oxidatively folded using the conditions optimized for the formation of linaclotide disulfide bonds.^35^ Peptides were dissolved in oxidation buffer (100 mM NaH_2_PO_4_, 2 M Gdn·HCl, pH 7.0) at ∼200 μM concentration and stirred for 24 h. Oxidation was monitored by analytical RP-HPLC and disulfide-bond formation confirmed by electrospray ionization mass spectrometry (ESI-MS). One major product was obtained. [Sec^1,6^; d-Tyr^14^]-Linaclotide was folded in 0.1 M ammonium bicarbonate buffer (pH 8.2, 100 μM peptide concentration, 6 h, 25 °C). Folding was accelerated due to the directed folding of the diselenide bond, which formed immediately after HF cleavage.^46,47^ After folding was complete, the pH was adjusted to 2 with neat TFA to stop the reaction, and the peptides were purified by preparative RP-HPLC to >95% purity.

### Synthesis of [Cyclic]-Linaclotide

The peptide was assembled on a 2-chlorotrityl hydrazine resin by Fmoc-SPPS. To prepare the 2-chlorotrityl hydrazine resin, the 2-chlorotrityl chloride resin was swelled in 50% (v/v) DMF/DCM and treated with 10% (v/v) hydrazine hydrate/DMF (2 × 30 min). The unreacted resin was deactivated with 5% (v/v) MeOH/DMF (10 min).^48,49^ Fmoc-Tyr(tBu) was activated with HCTU/DIPEA (1:1:1) and coupled to the resin (4 equiv). Resin loading capability was quantified by the molar difference between the Tyr-hydrazide resin and the original 2-chlorotrityl resin. NCL and oxidative folding were achieved by a one-pot reaction. Reduced [cyclic]-Linaclotide was dissolved in 0.2 M sodium phosphate/6 M GdnHCl (pH 3.0; 1 mM) and reacted with NaNO_2_ (10 equiv) for 15 min at −15 °C. MESNA (40 equiv) was added to the mixture, and after 5 min, the solution was diluted to ∼200 μM with oxidation buffer (100 mM NaH_2_PO_4_, 2 M Gdn·HCl, pH 7.0). After folding was complete, the pH was adjusted to 2 with neat TFA and [cyclic]-Linaclotide was purified by preparative RP-HPLC to >95% purity.

### RP-HPLC and LC–MS

Peptides were purified using a preparative C_18_ column (Eclipse XDB, Agilent; 10 μm, 21.2 cm × 250 mm, 80 Å, flow rate of 15 mL/min) in a Waters Delta 600 HPLC system (Waters Co., Milford, MA) with a gradient of 15–45% B over 60 min. Solvents consisted of 0.05% TFA in water (solvent A) and 90% ACN/0.043% TFA/10% H_2_O (solvent B). The molecular weight of the fractions collected was analyzed by direct injection on ESI-MS. Fractions with the desired mass were further analyzed by analytical reversed-phase (RP) high-performance liquid chromatography (HPLC) and lyophilized.

Analytical RP-HPLC on an analytical C_18_ column (Zobrax 300SB, Agilent; 3.5 μm, 2.1 × 200 mm, 300 Å, flow rate 0.3 mL/min) connected to a Shimadzu LC-20AT solvent delivery system equipped with a SIL-20AHT autoinjector and an SPD-20A Prominence UV–vis detector was used to monitor reactions and determine the purity of purified fractions/products. A linear gradient of 0–50% B over 50 min was used, and absorbance data were collected at 214 nm. Mass analysis was performed using a QStar Pulsar mass spectrometer (SCIEX, Ontario, Canada) with a Series 1100 solvent delivery system equipped with an autoinjector (Agilent Technologies Inc., Palo Alto, CA) and a C_18_ column (Phenomenex Jupiter, 90 Å, 4 μm, 250 mm × 2 mm). Linear gradients of 0.1% aqueous formic acid (solvent A) and 90% ACN/0.1% formic acid (solvent B) were used at a flow rate of 250 μL/min, and the column was kept at 45 °C. The instrument was scanned from 500 to 1800 *m*/*z*. Data acquisition and processing were carried out using Analyst software v1.1 (SCIEX, Canada).

### NMR

NMR spectra (1D ^1^H, 2D ^1^H–^1^H total correlation spectroscopy (TOCSY; 80 ms mixing time) and nuclear Overhauser effect spectroscopy (NOESY; 200 ms mixing time)) of peptides (1 mg) dissolved in 500 μL of 90% H_2_O/10% D_2_O were acquired using a Bruker 600 MHz Avance III NMR spectrometer equipped with a cryogenically cooled probe (cryoprobe) at 298 K. Samples were internally referenced to water at 4.76 ppm. TopSpin (Bruker Biospin) was used to process the spectra.

### Stability Assays

Simulated gastric fluid (SGF) was prepared by dissolving 20 mg of NaCl and 8 mg of pepsin in 70 μL of concentrated HCl (32%), and the volume was diluted to 10 mL with water (pH 1.3).^36^ Simulated intestinal fluid (SIF) was prepared by dissolving 68 mg of KH_2_PO_4_ in 500 μL of water followed by the addition of 800 μL of 0.2 M NaOH and 100 mg of porcine pancreatin, and the volume was adjusted to 10 mL with water (pH 6.8).^36^ Peptide stock solution (2 mg/mL in water, 15 μL) was diluted in 285 μL of SGF or SIF and incubated at 37 °C. Aliquots (30 μL) were withdrawn at 0 min, 5 min, 15 min, 30 min, 1 h, 2 h, 4 h, and 8 h and subsequently quenched with 30 μL of 0.2 M Na_2_CO_3_ (SGF) or 10% aqueous TFA (SIF). The samples were centrifuged, and the supernatant (15 μL) was analyzed by analytical HPLC. The remaining peptide was determined by measuring the peak area and expressing it as a percentage of the peak area at the 0 h time point. Peptide half-life (*t*_1/2_) was determined from the degradation profiles and calculated using Prism 7 (GraphPad, La Jolla USA), assuming an exponential one-phase decay.

### Cisbio cGMP Assay

Human epithelial colorectal adenocarcinoma T84 cells (ECACC) were routinely cultured in the DMEM + Ham’s F-12 medium supplemented with 10% heat-inactivated fetal calf serum and 2 mM l-glutamine at 37 °C in 5% CO_2_. Assays measuring cGMP accumulation were performed following the manufacturer’s instructions (cGMP HTRF assay kit, Cisbio International). In brief, increasing concentrations of linaclotide analogues were added to 20,000 cells in DMEM/F12 media containing 0.5 mM IBMX in a white 384-well plate (Optiplate, PerkinElmer Life Sciences). The plates were incubated for 30 min at 37 °C with 5% CO_2_. Cells were then lysed by the addition of HTRF reagents, the anti-cGMP-Eu-cryptate antibody, and the d2-labeled cGMP analogue diluted in lysis buffer (CGMP HTRF kit, Cisbio International) followed by incubation for 1 h at 25 °C. The emission signals were measured at 590 and 665 nm after excitation at 340 nm using a Tecan multilabel plate reader (Thermo Fisher Scientific).

## Supporting Information Available

Analytical HPLC, high-resolution MS traces, and 1D ^1^H NMR spectra of the linaclotide analogues (PDF)
Molecular formula strings (CSV)

## Abbreviations

ACN: acetonitrile
cGMP: cyclic-guanosine-3′,5′-monophosphate
DIPEA: *N*,*N*-diisopropylethylamine
DMEM: Dulbecco’s modified Eagle’s medium
FBS: fetal bovine serum
FDA: Food and Drug Administration
Fmoc-SPPS: 9-fluorenymethyloxycarbonyl-solid phase peptide synthesis
GC-C: guanylate cyclase-C
IBS: irritable bowel syndrome
MESNA: sodium 2-mercaptoethanesulfonate
NOESY: nuclear Overhauser effect spectroscopy
SGF: simulated gastric fluid
SIF: simulated intestinal fluid
Sta: heat-stable enterotoxin
STF-1: sunflower trypsin inhibitor 1
TOCSY: total correlation spectroscopy
TCEP: tris(2-carboxyethyl)phosphine
TIPS: tri-isopropylsilane
