# Supplementary material for: Improving the Gastrointestinal Stability of Linaclotide
Source: J Med Chem. 2021 May 12;64(12):8384–90. doi: 10.1021/acs.jmedchem.1c00380 (PMC8237258; doi:10.1021/acs.jmedchem.1c00380)
Supplement: Supplementary file 1 — jm1c00380_si_001.pdf [file jm1c00380_si_001.pdf]

Supporting Information

Improving the gastrointestinal stability of linaclotide

Nayara Braga Emidio[1], Hue N. T. Tran[1], Asa Andersson[1], Philip E Dawson[2], Fernando Albericio[3], Irina Vetter[1,4], Markus Muttenthaler*[1,5]

[1] Institute for Molecular Bioscience, The University of Queensland, Brisbane, Queensland 4072, Australia.

[2] Department of Chemistry, The Scripps Research Institute, La Jolla, California 92037, United States

[3] CIBER-BBN, Networking Centre on Bioengineering, Biomaterials and Nanomedicine, and Department of Organic Chemistry, University of Barcelona, 08028 Barcelona, Spain.

[4] School of Pharmacy, Pharmacy Australia Centre of Excellence, The University of Queensland, Woolloongabba, QLD, 4102 Australia.

[5] Institute of Biological Chemistry, Faculty of Chemistry, University of Vienna, 1090 Vienna, Austria.

***Corresponding Author**

Markus Muttenthaler – Institute for Molecular Bioscience, The University of Queensland, St Lucia, QLD 4072, Australia; Institute of Biological Chemistry, Faculty of Chemistry, University of Vienna, 1090 Vienna, Austria; Phone: (+43) 1 4277 70515; Email: markus.muttenthaler@univie.ac.at

Table of Contents

Figure S-1. Analytical HPLC and high-resolution MS traces of the linaclotide analogues S3

Figure S-2. Comparison of the 1D ^{1}H NMR spectra of linaclotide and its analogues S4

Figure S-3. Comparison of the 1D ^{1}H NMR spectra of [cyclic]-Linaclotide and linaclotide S4

Table S-1. List of calculated and observed masses S3

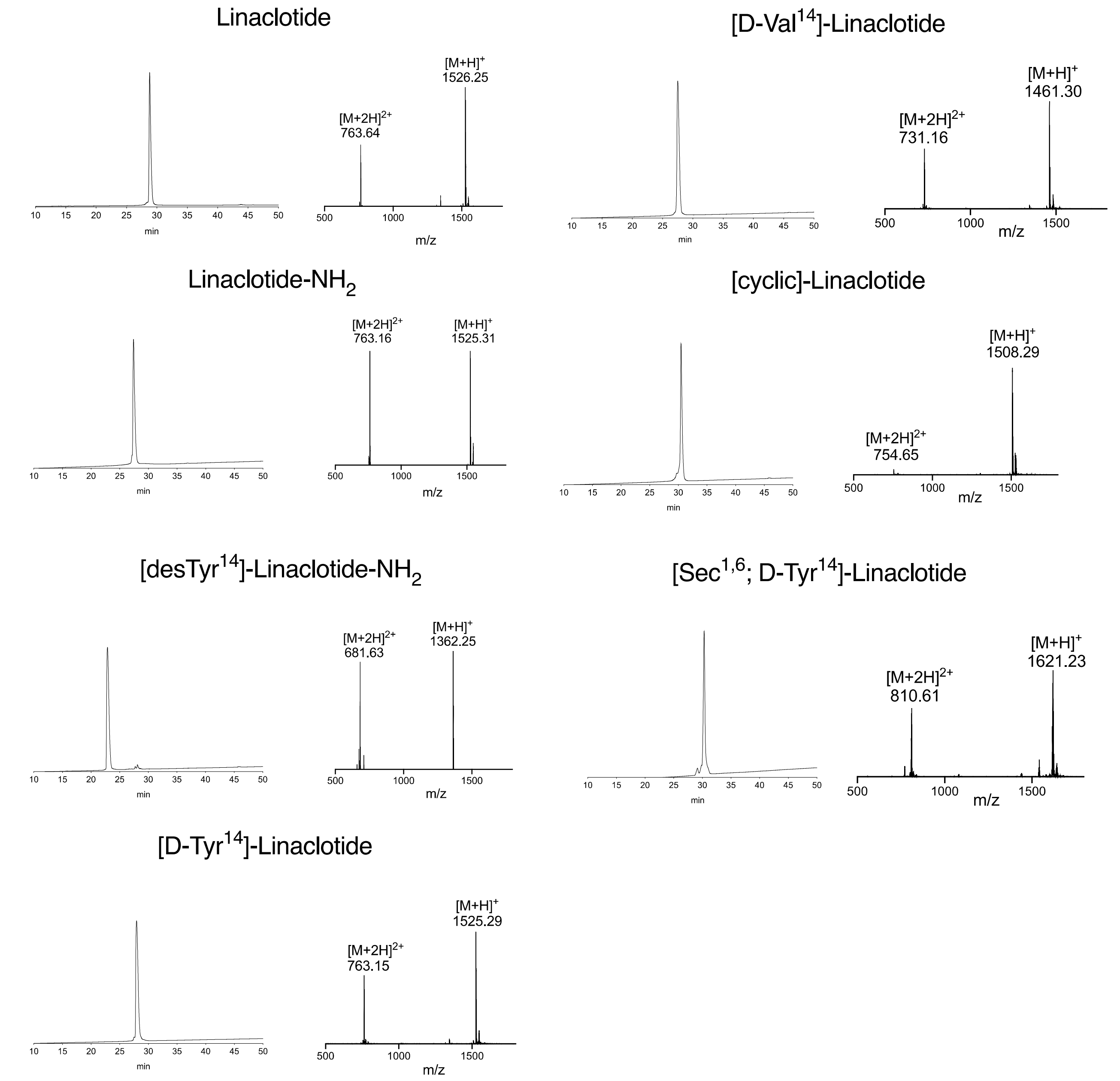

Figure S-1. Analytical HPLC and high-resolution MS traces of the linaclotide analogues.

Table S-1. List of calculated and observed masses.

Peptide	monoisotopic $[M+H]^+$ calc. mass	monoisotopic $[M+H]^+$ obs. mass
Linaclotide	1526.40	1526.25
Linaclotide-NH_2	1525.41	1525.31
[desTyr^{14}]-Linaclotide-NH_2	1362.35	1362.25
[D-Tyr^{14}]-Linaclotide	1525.41	1525.29
[D-Val^{14}]-Linaclotide	1461.42	1461.31
[cyclic]-Linaclotide	1508.39	1508.29
[Sec^{1,6}; D-Tyr^{14}]-Linaclotide	1621.30	1621.23

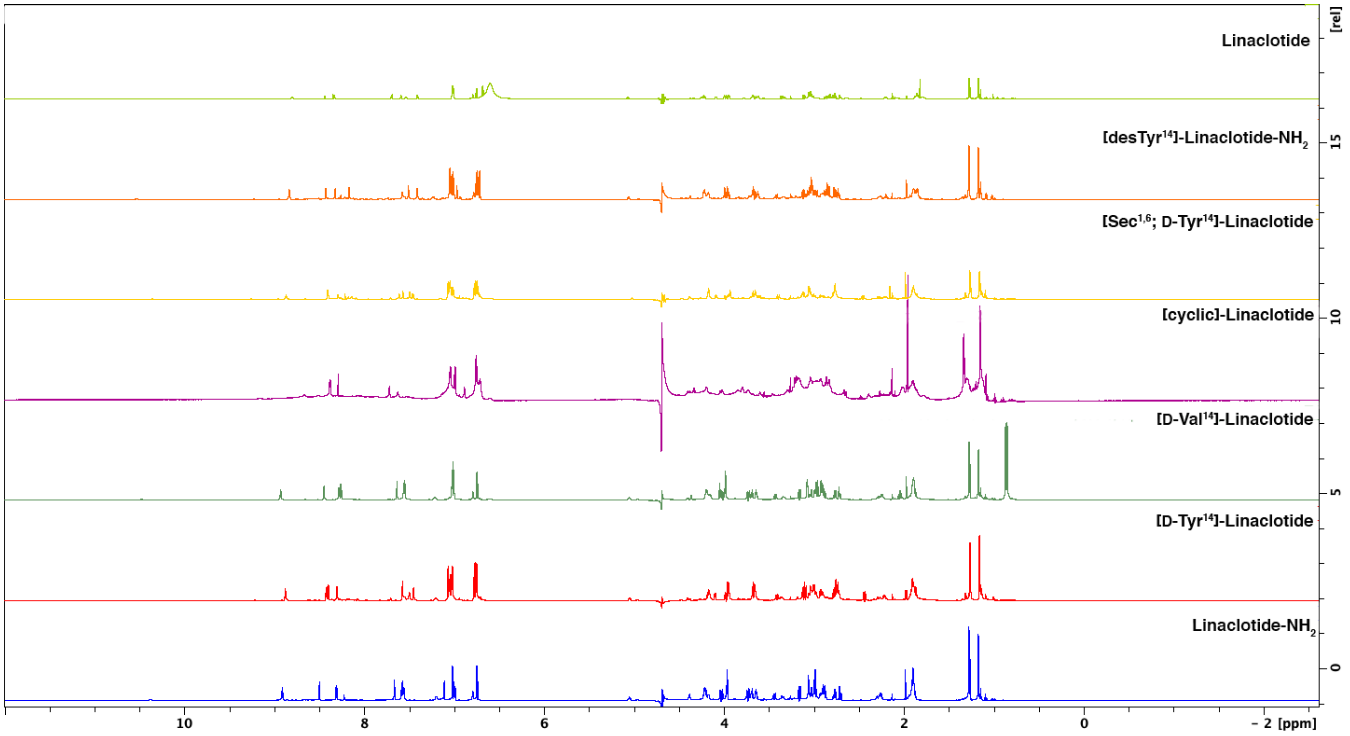

Figure S-2. Comparison of the 1D ^{1}H NMR spectra of linaclotide and its analogues.

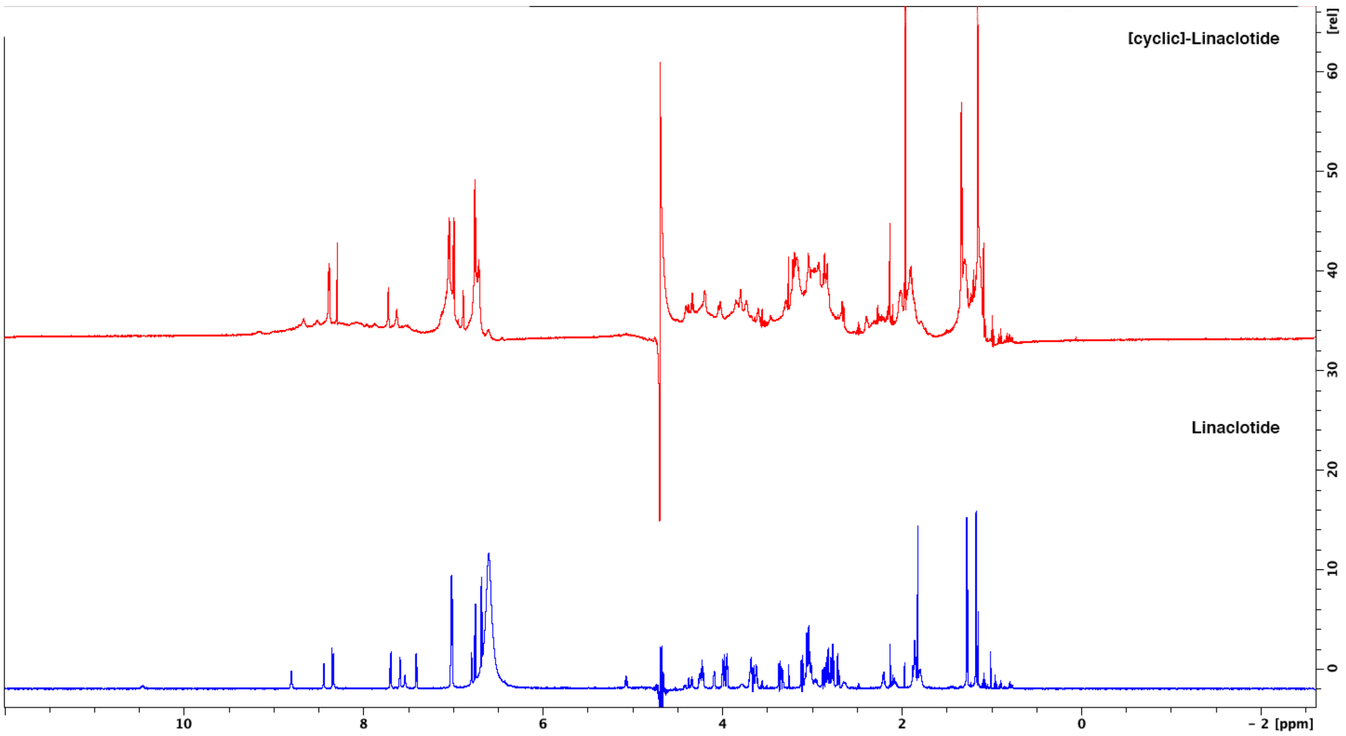

Figure S-3. Comparison of the 1D ^{1}H NMR spectra of [cyclic]-Linaclotide and linaclotide.
